# Removal of the Water Pollutant Ciprofloxacin Using Biodegradable Sorbent Polymers Obtained from Polysaccharides

**DOI:** 10.3390/polym15153188

**Published:** 2023-07-27

**Authors:** Sarah Alvarado, Alicia Megia-Fernandez, Mariano Ortega-Muñoz, Fernando Hernandez-Mateo, F. Javier Lopez-Jaramillo, Francisco Santoyo-Gonzalez

**Affiliations:** 1 Department Organic Chemistry, Faculty of Sciences, University of Granada, 18073 Granada, Spain; 2 Unit of Excellence in Chemistry Applied to Biomedicine and the Environment, University of Granada, 18073 Granada, Spain; 3 Biotechnology Institute, University of Granada, 18071 Granada, Spain

**Keywords:** cross-linking, ciprofloxacin, divinyl sulfone, emerging pollutant, biodegradable polymers, sorbent material, water management

## Abstract

Water use has been increasing globally by 1% per year, and recycling and re-use are critical issues compromised by the presence of pollutants. In this context, the design of novel materials and/or procedures for the large scale-removal of pollutants must be economically and environmentally feasible in order to be considered as part of the solution by emerging economies. We demonstrate that the cross-linking of biodegradable polysaccharides such as starch, dextrin, or dextrin and β-cyclodextrin with divinyl sulfone is an innovative strategy for synthesizing insoluble and eco-friendly sorbent polymers, including **pSt**, **pDx** and **pCD-Dx**. The evaluation of these polymers’ ability to remove ciprofloxacin (CIP), a prime example of antibiotic pollution, revealed that **pSt**, with a Kd of 1469 L/kg and a removal rate higher than 92%, is a favorable material. Its sorption is pH-dependent and enhanced at a mildly alkaline pH, allowing for the desorption (i.e., cleaning) and reuse of **pSt** through an environmentally friendly treatment with 20 mM AcONa pH 4.6. The facts that **pSt** (i) shows a high affinity for CIP even at high NaCl concentrations, (ii) can be obtained from affordable starting materials, and (iii) is synthesized and regenerated through organic, solvent-free procedures make **pSt** a novel sustainable material for inland water and seawater remediation, especially in less developed countries, due to its simplicity and low cost.

## 1. Introduction

As a result of a growing population, economic development, and consumption patterns, global water use has increased during the last century by a factor of six, at a rate of 1% per year, and water-stressed regions are distributed across every continent [1]. Water recycling and re-use are becoming critical issues in addressing water scarcity (i.e., water demandsexceeding the available supply), and water management is a current challenge. The presence of pollutants may increase the cost of water treatment and compromise water re-use, especially when considering that the majority of this increase in water consumption is concentrated in middle- and lower-income countries.

Pharmaceuticals are emerging as rapidly growing environmental contaminants that are present in nearly all matrices, including the polar regions, which are considered as the most pristine environment [2]. Although the presence of pharmaceuticals in rivers [3] and in irrigation waters [4] poses a serious global threat to human health and the environment, they are still unregulated [5]. As emerging pollutants, they represent a challenge for water treatment and are an international research focus [6].

Among environmental pharmaceuticals, antibiotics are a major concern because they may promote antimicrobial resistance (AMR) in microorganisms and contribute to the global AMR crisis [7]. Quinolones are an illustrative example of antibiotic pollution because they are broad-spectrum antibiotics that are used in both human medicine and livestock farming [8,9]. In the case of many quinolones (many of them are not metabolized), the metabolites are excreted via urine and feces, and significant amounts of these quinolones have been detected in urban and hospital wastewater, effluents from wastewater treatment plants (WWTPs), sediments, freshwater and saltwater bodies [8,9]. Additionally, some quinolones have been found in livestock and wildlife, whereas others have the capacity to spread from soil to cultivated plants like radishes, beans, lettuces, or cucumbers, and others have been detected [9,10]. 

Ciprofloxacin (CIP) is a prime example of the quinolone concern and a real environmental issue. Although, as a drug used in human medicine, CIP is not permitted for livestock farming, it is a metabolite of enrofloxacin, a fluoroquinolone for exclusive use inthe farming industry, and levels as high as 43 µg/kg have been reported in samples of horse manure [11]. In fact, CIP has been detected in different rivers [3], and levels of 31 mg/L (corresponding to a daily release of 44 kg, sufficient to treat 44,000 patients) have been reported in the effluents of pharmaceutical plants in India [12].

Different materials have been studied for the removal of CIP from water, including carbon adsorption, nanoparticles, chemical oxidation, advanced oxidation processes, or electrochemical oxidation [6]. Although feasible, many approaches may be compromised from a practical standpoint by the cost of implementation, and sorption has been suggested as a more promising alternative. In particular, the list of sorbents reported to remove CIP includes biochar [13,14,15], biosorbents [16,17], nitrocellulose [18], lignin-based sorbents [19], activated carbons, bauxite [20], and carbon nanotubes [21].

Polysaccharides have garnered significant interest in the design of sorbent materials due to their good sorption performance, low cost, biodegradability, environmental friendliness, and renewability. In the context of wastewater treatment, polysaccharide-based sorbents have been obtained through the chemical or physical cross-linking of different polysaccharides [22,23]. In particular, CIP sorption on chitosan-based materials [24], magnetized cross-linked maltodextrin [25], carrageenan-coated magnetic nanoparticles [26], and cross-linked cyclodextrin [27] have been reported. 

**Scheme 1:**
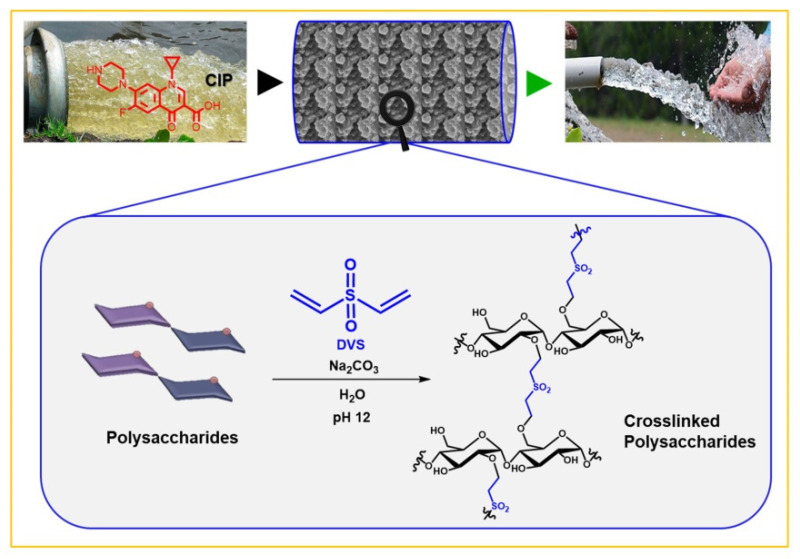
Cross-linking of polysaccharides with divinyl sulfone in water yields a reusable polymer that removes 92% of ciprofloxacin from water, even at high NaCl concentrations.

Bearing in mind the importance of water re-use and the fact that the large-scaleremoval of water pollutants requires materials and/or procedures that must be economically and environmentally feasible, we hypothesize that the cross-linking of biodegradable starting materials leads to the reticulation and formation of cavities with the ability to sorb different molecules. Herein, we report on the synthesis and characterization of sorbent materials obtained through the reaction of starch (St), dextrin (Dx) or β-cyclodextrin (β-CD) and Dx with the cross-linker divinyl sulfone (DVS) to yield the homopolymers **pSt** and **pDx** and the heteropolymer **pCD-Dx**. As a promising material for the removal of CIP from aqueous matrices, **pSt** was the focus of our research (Scheme 1).

## 2. Materials and Methods

### 2.1. Reagents

Research grade native β-cyclodextrin (β-CD, 98%, sum of other cyclodextrin-related impurities ≤0.5%, Cyclolab, Budapest, Hungary), soluble potato starch (St, residue after ignition 0.3%, Sigma-Aldrich, Saint Louis, MO, USA) and dextrin from potato starch (Dx, Fluka, Saint Louis, MO, USA), divinyl sulfone (DVS, 99.5%, TCI, Zwijndrecht, Belgium), ciprofloxacin [1-Cyclopropyl-6-fluoro-4-oxo-7-(piperazin-1-yl)-1,4-dihydro-quinoline-3-carboxylic acid] (CIP, 98%, TCI, Zwijndrecht, Belgium) and ofloxacin [(RS)-9-Fluoro-2,3-dihydro-3-methyl-10-(4-methylpiperazin-1-yl)-7-oxo-7H-pyrido [1,2,3-de]-1,4-benzoxazine-6-carboxylic acid] (OFL, 98%, BDLpharm, Kaiserslautern, Germany) were used as received. Anhydrous sodium carbonate (99.5%), anhydrous sodium acetate (99%), and 4-(2hydroxyethyl)-1-piperazineethanesulfonic acid (HEPES, 99.5%) were purchased from Sigma-Aldrich (Saint Louis, MO, USA).

### 2.2. Synthesis of Cross-Linked Polymers

Cross-linked polymers were obtained, as previously reported [28,29]. In brief, Dx or St (50 g), or a mixture of β-CD (25 g) and Dx (25 g), was dissolved in carbonate buffer (3 M, pH 12, 500 mL) with magnetic stirring at room temperature. For the particular case of St, heating to reflux was required for the complete solubilization, and it remained stable when brought to room temperature. The solution was stirred for 30 min, and then a volume of 25 mL of DVS (28.5 g, 0.242 mol) was added drop-wise while stirring. The cross-linking reaction was allowed to proceed overnight under stirring. Cross-linked polymers **pSt**, **pDx**, and **pCD-Dx** appeared as a precipitate that was isolated by filtration, thoroughly washed first with deionized water, then with methanol, and finally with diethyl ether. After drying under vacuum for 18 h at 40 °C, the obtained amounts of **pSt**, **pDx**, and **pCD-Dx** were 52.9 g, 40.5 g, and 34.5 g, respectively.

### 2.3. Characterization

Cross-linked polymers were characterized by elemental analysis with a Thermo Scientific Flash 2000 elemental analyzer (Thermo Scientific, Waltham, MA, USA) to determine the presence of S from the sulfone group of the DVS cross-linker. Structural characterization of the polymers was carried out by Fourier transform spectroscopy (FT-IR) and X-ray powder diffraction (XRPD). IR spectra from 400 to 4000 cm^−1^ were measured with a Spectrum Two FT-IR spectrometer (PerkinElmer, Waltham, MA, USA) in ATR mode by accumulating 30 scans. X-ray diffractograms were collected with a D8 Discover equipped with a Pilatus3R 100K-A detector (Bruker, Billerica, MA, USA), operation voltage and current of 50 kV and 1 mA, respectively, and Cu Kα (λ = 1.54 Å) sealed tube. Data were collected from 2θ 6° to 70° with a 0.02° step and 40 s of integration time and analyzed with Xpowder software (2004.04.49) [30].

Polymer **pSt** was further characterized by thermogravimetric analysis (TGA) to study the thermal stability and identify the products of decomposition and by electron microscopy to observe the morphology. TGA was performed in a nitrogen atmosphere at 950 °C and heating rate of 20 °C/min using a Shimadzu TGA-50H instrument (Shimadzu, Kyoto, Japan) coupled to a Nicolet 550 IR-FT spectrometer (Thermo Scientific). For the electron microscopy analysis, the sample was covered with gold using a sputter coater (SEMPrep2, Technoorg Linda LTD, Budapest, Hungary) and analyzed with a Hitachi S-510C scanning electron microscope at 3 kV (Hitachi High Technologies Europe GmbH, Krefeld, Germany).

### 2.4. Sorption Studies

All sorption experiments were conducted at room temperature in batch mode with 0.1 g of cross-linked polymer and 10 mL of fluoroquinolone (i.e., CIP or OFL) water solution (concentration range from 0.01 to 0.2 mg/L). The experiments were carried out in Falcon tubes, and the suspensions were mixed in a tube rotator (VWR) for 3 h. Then, the quinolone solution was separated by centrifugation at 4000 rpm, and its concentration was quantified with an F2000 fluorescence spectrophotometer (Hitachi, Tokyo, Japan) by interpolating the emission at 452 nm (λex 273 nm) in a calibration curve.

The ability of cross-linked polymers to sorb CIP and OFL was evaluated by the sorption coefficient, Kd, which is defined as the ratio between the concentration of the fluoroquinolone in solution (Ce) and in the polymer (qe) and is estimated as the slope of the plot qe (mg/kg) vs. Ce (mg/L) at equilibrium [21]

Kd = dqe/dCe

### 2.5. Modeling of Sorption Experiments

Data fitting was carried out with ISOT-Calc, a macro for MS-Excel that performs a non-linear regression to distinct isotherms, being the minimization of the mass balance (i.e., the difference between the estimated and the experimental Ce values) the objective function(U) [31]. *U* is defined as the sum of squared residual errors (*e_i_*) obtained from the difference between the experimental and the corresponding values estimated by the guessed model, with *w_i_* being statistical weights:

$$U=\sum_{i=1}^{n}w_{i}\cdot e_{i}^{2}$$

Data were fitted to the two parameters isotherms of Langmuir, Freundlich, and Temkin, the three parameters isotherms of Redlich–Peterson and Vieth–Sladek, and the four parameters isotherm of 2-sites Langmuir as defined by ISOT-Calc and depicted in Table S1 [31]. The goodness of the fitting was judged by evaluating the standard deviation of the parameters defining the isotherm and the mean weighted squared error (MWSE) defined as

$$\mathrm{MWSE}=\frac{U}{\left(n-p\right)}$$

where *n* indicates the number of experimental points and *p* the number of refined parameters.

### 2.6. Effect of the Ionic Strength and pH on the Sorption of CIP onto pSt

An amount of 50 mg of **pSt** was incubated in a Falcon tube with 9 mL of a 20 mg/mL CIP solution supplemented with 1 mL of buffering solution (sodium acetate pH 4.6 or 6, HEPES pH 7 or 8, Tris-HCl pH 8 or carbonate pH 9.5) to provide a buffer concentration of 10 mM, 25 mM or 50 mM and pH values of 4.6, 6.0, 7.0, 8.0, and 9.5. Suspensions were mixed in a tube rotator (VWR, Radnor, PA, USA) for 3 h. Then, the quinolone solution was separated by centrifugation at 4000 rpm, and its concentration was quantified with an F2000 fluorescence spectrophotometer (Hitachi) by interpolating the emission at 452 nm (λex 273 nm) in a calibration curve.

The effect of high concentrations of NaCl was evaluated on 100 mg of **pSt** and 0.2 mg/mL CIP in either water or 10 mM HEPES pH 8. The solutions were supplemented with NaCl to a final concentration of 4‰, 14‰, and 24‰ (brackish water); 35‰ (seawater); and 50‰ (brine), and the experiment proceeded as described above.

### 2.7. Fixed Bed Studies with pSt

An amount of 0.5 g of **pSt** was packed into a 2.5 mL syringe, and a solution of either 10 mg/L or 200 µg/mL of CIP in water was flown through at 1.7 mL/min with the help of a peristaltic pump (MasterFlex, Cole Parmer, Vernon Hills, IL, USA) and passed through a fluorescence cell located into an F2000 fluorescence spectrophotometer (Hitachi) to record the fluorescence at 452 nm (λex 273) (Figure S1). The polymer was regenerated by pumping 20 mM AcONa pH 4.6.

## 3. Results

In the environmental field, there is a need for creating new polymeric materials, modifying existing polymers, and discovering green and novel applications of conventional polymers. In this context, bio-polymers have attracted attention as eco-friendly alternatives to petroleum-based materials, and we hypothesized that the cross-linking of carbohydrates yields the reticulation and formation of new polymeric materials with cavities that may resemble the ability of cyclodextrins to form inclusion complexes with different molecules.

We have focused our study on soluble starch (St), dextrin (Dx), and β-cyclodextrin (β-CD). St attracted our attention because it is the least expensive polysaccharide, it can be obtained from abundant and widely distributed plants, and St has already been used in advanced functional material applications, including water treatment [32,33]. The major drawback of St is its water insolubility which can be partially overcome by using soluble starch, although it implies an extra cost derived from the use of high temperature to yield the complete dissolution of the high concentration of St needed for the cross-linking. An alternative is the use of more soluble oligosaccharides. For this reason, we have also evaluated both the linear oligosaccharide Dx and its combination with the cyclic oligosaccharide β-CD since the potential of CD-based copolymers and CD-containing polysaccharides in water remediation has been explored [23].

### 3.1. Synthesis and Characterization

Our group has assessed that the oxo-Michael addition of polysaccharides to divinyl sulfone (DVS) in aqueous media yields insoluble materials with the capacity to sorb phenolic pollutants and bioactive compounds [28,29]. This is a versatile, eco-friendly, and economically affordable strategy that can be implemented and scaled up in developing countries where water pollution is a first-order problem [34]. In this context, St, Dx, and a mixture of Dx and β-CD were reacted with DVS in carbonate buffer pH 12 to produce insoluble materials corresponding to the homo cross-linked polymers **pSt** and **pDx**, and the hetero cross-linked polymer **pCD-Dx**, which were isolated by filtration, thoroughly washed and dried in vacuo with yields (i.e., percentage of the mass of reactants recovered as an insoluble polymer) of 67.4%, 51.6%, and 43.9%, respectively. Elemental analysis found more than 7% of sulfur in these polymers (Table S2). Considering that each molecule of cross-linker consists of four C and one S and that each molecule of glucose contains six C, the ratio Glc/DVS was estimated as 1.7 for **pDx** and **pCD-Dx**, and 1.9 for **pSt**, confirming the success of the cross-linking reaction.

**Figure 1:**
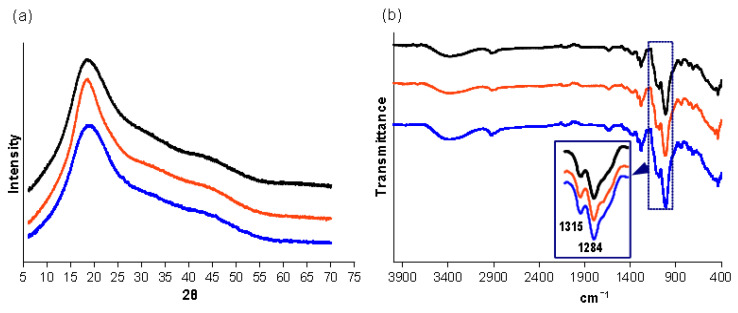
XRD (**a**) and FTIR (**b**) of **pSt** (blue), **pDx** (black), and **pCD-Dx** (red).

These polymers were characterized by XRD and FTIR. The XRD analysis reveals only dispersive broad peaks centered at 2θ 18.5° for **pDx** and **pCD-Dx** and 20.0° for **pSt** (Figure 1a). These results were anticipated because the diffractograms of the starting materials St and Dx show poor crystallinity (Figure S2). Moreover, the decrease in the crystalline features of β-CD is expected due to the formation of a hybrid polymer (i.e., **pCD-Dx**) and new covalent bonds. The FTIR spectra of the insoluble polymers show the broad signal of the O-H stretching at 3500 cm^−^^1^ and a double signal at 1284 and 1315 cm^−^^1^ that matches with the distinctive signature of the sulfone group [35], further confirming the cross-linking (Figure 1b).

### 3.2. Characterization of the Polymers as Sorbents for CIP and OFL

Global development has led to a six-fold increase in the use of water with the concomitant emergence of water-stressed regions in every continent [1]. Water management is a current challenge, and water recycling and reuse is a feasible alternative to address the water scarcity that, however, is hampered by the presence of water pollutants. Quinolones are an illustrative case of water pollutants since they are used in both human medicine and livestock farming, and a significant amount of them have been detected in urban and hospital wastewater, waste from pharmaceutical plants and water treatment plants, as well as in sediments and fresh and saltwater bodies [8]. Fluoroquinolones are good targets to assess the efficiency of **pSt**, **pDx**, and **pCD-Dx** as sorbents, and we focussed on CIP because, in addition to being a human medication, it is also a metabolite from enrofloxacin, which is used in the farm industry. As a result, CIP is frequently detected in rivers in Africa, Asia, and Europe, with concentrations exceeding the safe values in a significant percentage of cases [3]. Preliminary experiments demonstrated that the incubation of 0.1 g of the cross-linked polymers **pSt**, **pDx**, and **pCD-Dx** with 10 mL of CIP solutions at concentrations ranging from 10 µg/L to 250 µg/L reached the equilibrium at 2.5 h and that 10 µg/L is the limit for a reliable quantification by fluorescence of the distribution of CIP between the water and the polymer. Consequently, these were the conditions of the assay, but with the incubation time extended to 3 h. Additionally, we also conducted a preliminary exploration of the ability of these polymers to remove OFL from aqueous solutions because OFL, as CIP, is widely used in medicine and is reported as a water pollutant [4,36]. Our results show that **pSt** removes the largest percentage of these drugs from the solution and also reveal a linear relationship between the amount of quinolone retained by the polymers and the initial concentration (Figure S3). This result was unexpected because, unlike **pSt** and **pDx**, where cavities result from the cross-linking, those in **pCD-Dx** include the pre-formed cavity of β-CD, which has been reported to host and form inclusion complexes with both CIP and OFL [37].

### 3.3. Study of the CIP and OFL Sorption on pSt

**Figure 2:**
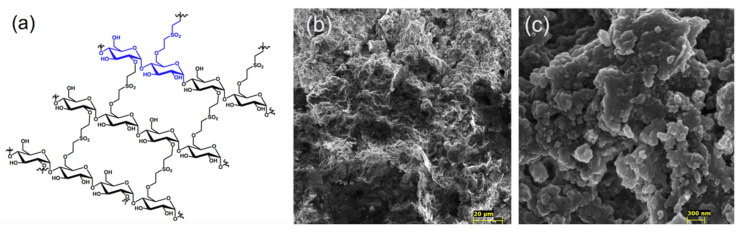
Model of the structure (**a**) and SEM micrographs of **pSt** at 1.23 K x (**b**) and 50.00 K x (**c**) magnification (scale bars 20 µm and 300 nm, respectively)**.** The repeating unit is shown in blue.

In order to explore the good performance of **pSt**, a more exhaustive characterization was addressed. Assuming from the elemental analysis that the ratio of Glc/DVS is 1.9, we hypothesize that the repeating unit of **pSt** comprises two molecules of glucose and one of DVS and that a plausible structure of **pSt** resulting from the reticulation would produce cavities consisting of two dimers of Glc cross-linked by two molecules of DVS (Figure 2a). These cavities may resemble the features of β-CD and be more suitable to host CIP and OFL, supporting the good performance of **pSt**. SEM analysis of **pSt** reveals a rough surface that, at higher magnification, presents a microgranular aspect (Figure 2b,c), which is completely different from the globular appearance of **pCD-Dx** (Figure S4).

**Figure 3:**
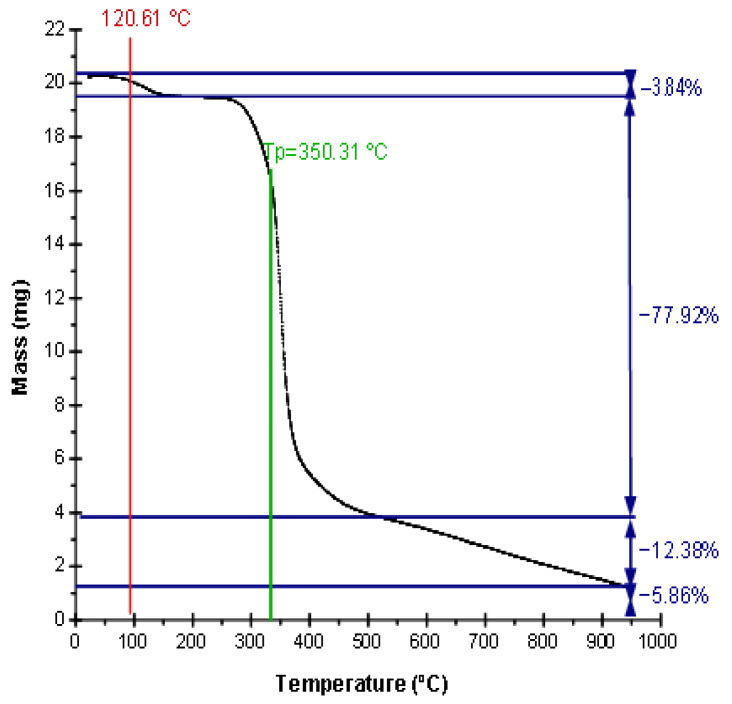
TGA thermal curve of **pSt** showing the percentage of weight loss (blue) and the 1st derivative peaks (red and green).

TGA-IR analysis was conducted for **pSt** (Figure 3 and Figure S5). When the sample is heated to 950 °C in a nitrogen atmosphere at a heating rate of 20 °C/min, a first mass loss of 3.84% is detected, with a maximum speed of decomposition at 120.6 °C. This mass loss is within the typical range of values described for a hydrophilic polymer such as poly(vinyl acetate), and it is related to the vaporization of bound water [37]. A second mass loss of 77.9% takes place within the interval 250–550 °C, the onset temperature (To) being 325 °C with the highest speed of decomposition at 350.3 °C (Tp), and corresponds to depolymerization and decomposition of the polymer **pSt** and the structure of St. Both, To and Tp fall within the values published for polymers obtained by cross-linking of carbohydrates with DVS [28]. Beyond 550 °C, the degradation of the organic matter accounts for a 12.4% mass loss and leaves a residue as ash (5.9%). The IR spectra collected during the analysis allow the detection of signals assigned to CO_2_ (3734, 3626, 2357, and 2324 cm^−1^), CO (2176, 2113, and 666 cm^−1^), and SO_2_ (2515, 2486, 1375, 1359, 1340, 1166, and 1129 cm^−1^), as well as weak signals at 1749 and 1166 cm^−1^, 2986 and 1746, 3126 and 948, and 3015 cm^−1^ which may indicate the presence of formaldehyde, acetaldehyde, ethene, and methane, respectively (Figure S6).

**Figure 4:**
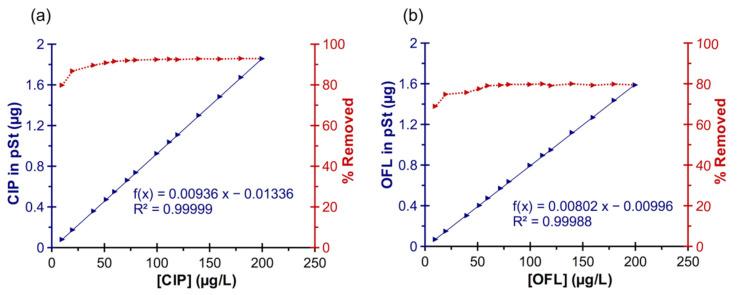
Sorption of CIP (**a**) and OFL (**b**) on 100 mg of **pSt**. Amount retained by the polymer (in blue) and percentage removed from the solution (in red) as a function of the initial concentration of quinolone.

The ability of **pSt** to remove CIP and OFL from an aqueous solution was evaluated by incubating 0.1 g of the polymer with 10 mL of 14 solutions with concentrations ranging from 10 to 200 µg/L. The amount of quinolone retained by the polymer and the initial concentration in the solution is linearly related, as predicted from the preliminary characterization. The extraction was performed consistently for the solutions with concentrations between 60 and 200 µg/L, with removal rates of more than 92% for CIP and 80% for OFL (Figure 4). Based on these data, the sorption coefficients of CIP and OFL were estimated to be 1469 L/kg and 405 L/kg, respectively, with coefficients of determination (R^2^) better than 0.99 (Figure S7). It has been stated that the extraction by sorption of pollutants in municipal WWTPs is negligible for Kd values lower than 500 L/kg [37] and, in the context of wastewater treatment, the Kd value of 1469 L/kg and a removal rate higher than 92% make **pSt** an attractive material for the removal of CIP from water.

The above fitting of the data to a “C” isotherm is not unexpected for pollutants in water, and it may be a consequence of the low concentration of the experimental data rather than an accurate description [38]. In order to gain additional understanding of the sorption process, data were fitted to the two parameters isotherms of Langmuir, Freund- lich, and Temkin, the three parameters isotherms of Redlich–Peterson and Vieth–Sladek and the four parameters isotherm of 2-sites Langmuir as defined in Table S1. Although the linearized forms of the isotherms have been extensively referenced in the literature, linearization implies bias, so our efforts were focused on non-linear fitting with the tool ISOT_Calc [31]. The goodness of the fitting was evaluated from the standard deviation (% r.s.d.) of the parameters defining the isotherm and the mean weighted squared error (MWSE). Fitting always converged in a solution for the different isotherms, with MSWE ranging from 10^−2^ to 10^−3^ (Table 1 and Table S3). However, when those with a % r.s.d. larger than 100% were discarded, only the isotherms of Freundlich and Temkin yielded a feasible fitting (Table 1). This coincidence was not unexpected since it has been reported that the Freundlich and Temkin isotherms are practically equivalent in terms of fitting ability [39].

Although sorption isotherms do not have any intrinsic thermodynamic definition and their significance depends on the conditions from which they were obtained [38], K_F_ of the isotherm of Freundlich has been associated with the sorption capacity and N to a heterogeneity parameter or, alternatively, to the strength of the process. When N is 1, the partition between the two phases is independent of the concentration (i.e., linear isotherm), while values larger than 1 are indicative of cooperative sorption [40]. The fact that K_F_ is two orders of magnitude larger for CIP is in agreement with the better performance of **pSt** in the removal of CIP. On the other hand, OFL N is very close to 1, in agreement with the zero-origin line fitting resulting from the estimation of the sorption coefficient. These results support that the sorption of CIP is better described by the isotherm of Freundlich, and the process may exhibit some cooperativity, whereas the sorption of OFL may be described as a C-isotherm.

### 3.4. Factors Affecting the Sorption of CIP and OFL on pSt

**Figure 5:**
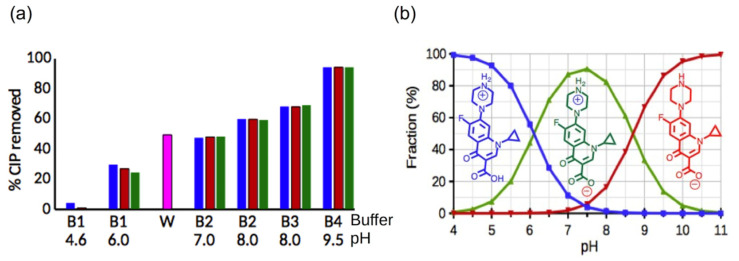
Influence of the pH and the ionic strength on the sorption of CIP on 50 mg of **pSt** (**a**) and distribution of the different species of CIP as a function of the pH of the solution, considering pKa1 = 6.1 and pKa2 = 8.7 (**b**). In (**a**), the buffering systems were 10 mM (blue), 25 mM (red), and 50 mM (green) of acetate (B1), HEPES (B2), Tris-HCl (B3), and carbonate (B4) at pH 4.6, 6.0, 7.0, 8.0, and 9.5. As a reference, a control experiment was carried out in water (W).

The nature of the interactions between the sorbent and the contaminant is an important aspect to consider in order to understand the efficiency of the sorption process. There exists abundant bibliography on the sorption of CIP on different materials. Hydrophobic interactions have been described in activated carbon [41] and biochar [13], whereas electrostatic forces have been reported in activated sludges [42], sandy and sandy clay loam soils [5], biosorbents [16], or lignin-based sorbents [19]. Since the CIP molecule forms a zwitterion resulting from ionization of the acidic group with a pKa of 6.1 and a basic group with a pKa of 8.7, pH and ionic strength are expected to condition the net charge of the molecule and its hydrophobicity, resulting in an alteration of the performance of **pSt**. Our results reveal a strong positive correlation with the pH, with the efficiency of the sorption increasing with the pH, whereas at acidic pH, the reduced sorption is negatively affected by the ionic strength (Figure 5a). Considering the distribution of species as a function of the pH and that the main species present at pH 5 is CIP^+^, at pH 9.5 is CIP^−^, and between pH 7 and 8 is CIP^±^ (Figure 5b), our results can be interpreted as that **pSt** is not efficient in trapping CIP^+^ and shows higher affinity for CIP^−^ than for CIP^±^. The fact that the ionic strength heavily influences the sorption at the pH range where the sorption is low but its effect is negligible at pH > 7 led us to hypothesize that the main driving force is the ionic interaction between the carboxylate group of CIP^−^ and **pSt** and, secondarily, a weaker interaction with the lone electron pair of the nitrogen in the piperazine ring linked to the quinolone. Thus, the weak interaction is primarily responsible for the sorption at acidic pH, where the carboxylate group is protonated and is disrupted by ionic strength. As the carboxylate is formed at high pH values, a strong interaction enters the scene, and the screening is negligible.

Our hypothesis of the interactions driving the sorption of CIP was put to the test with an experiment at a high concentration of NaCl (Figure S8). When the experiment was conducted in water, the sorption of CIP decreased from 95% at 0‰ NaCl to 78% at 50‰ NaCl. The effect of the ionic strength, while maintaining a linear relationship with the efficiency of **pSt**, is partially overcome by pH. Thus, although at 0‰ NaCl, the amount of CIP trapped by **pSt** is basically the same regardless of the pH, the slope of the linear fitting is four-fold higher for the experiments in water. It is important to highlight that CIP^±^ is the main species in both water (71%) and pH 8 (80%) and that the major difference between both conditions is the second most abundant species, which is CIP^+^ (28%) in water and CIP^−^ (17%) at pH 8 (Figure 5b). Thus, by increasing the pH, the low-affinity CIP^+^, whose weak interaction is heavily screened by ionic strength, virtually disappears and is replaced by the high-affinity CIP^−^, whose interaction with **pSt** is less dependent on the ionic strength. These findings not only support our hypothesis but also have practical implications since high-salinity wastewater accounts for 5% of the total amount of industrial sewage, and in some coastal areas, desalination is a source of water. In this context, **pSt** is an attractive material that works at 35‰ NaCl (seawater) and even at 50‰ NaCl (brine), whose performance can be enhanced by buffering at the mildly alkaline pH 8 (Figure S8).

### 3.5. Fixed Bed Studies with pSt

**Figure 6:**
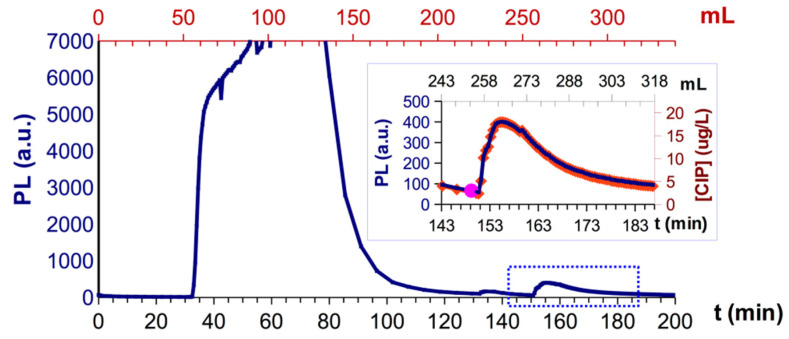
Chromatogram showing the evolution of the fluorescence eluted from 0.5 g of **pSt** packed into a syringe resulting from the passage of a solution of 10 mg/L CIP at a flow of 1.7 mL/min. Once saturated and washed with distilled water to remove the unbound CIP, the retained CIP was eluted with 20 mM AcONa pH 4.6 (Insert. The pumping of the AcONa solution starts at the magenta dot). The detector saturates at 7000 a.u.

In general, WWTPs are not designed to deal with complex chemical molecules such as pharmaceuticals, and new technologies are needed to remove them. However, cost is an important parameter to consider for their practical applications. The fact that typically sorption requires less implementation, the affordability and low cost of the starting materials, and the good results in batch experiments make **pSt** a promising material that encouraged us to study its performance as a fixed bed and the viability of recycling it. An amount of 0.5 g of **pSt** packed in a 2.5 mL syringe (Figure S1) was saturated by passing a solution of 10 mg/L CIP at a flow rate of 1.7 mL/min. After saturation of the polymer and passing distilled water to eliminate the unbound CIP, the low affinity of **pSt** for CIP^+^ was exploited to elute CIP and regenerate the polymer, allowing its recycling (Figure 6). Thus, conditions as eco-friendly as 20 mM acetate pH 4.6 transformed the bound CIP into CIP^+^, resulting in its release (Figure 6, insert). The regenerated column was then evaluated against a new 10 mg/L CIP solution, being observed that it was able to remove up to 99.5% of the CIP to yield an eluted solution with a concentration of CIP lower than 50 µg/L in the first 56 mL eluted (i.e., 37 column volumes), the extraction factor being estimated as 200 (Figure S9a). Once it was demonstrated that after recycling, **pSt** retains its ability to trap CIP, and after a new regeneration, it was further evaluated against a 200 µg/L CIP solution as a more realistic scenario (Figure S9b). Results show that the regenerated **pSt** maintains its high affinity and reduces the content of CIP down to 10 µg/L in 1.8 L of treated water (Figure S9b).

## 4. Conclusions

The increase in water consumption combined with climate change will aggravate the situation of water-stressed regions, and the presence of water pollutants represents an additional difficulty. Since sorption is a versatile, low cost and easy operating procedure, the incorporation of economically and environmentally feasible sorbent materials is a keystone in the strategies of water management. Those based on cross-linked polysaccharides are promising, and **pSt** fulfills both requirements. The starting materials, St and DVS, are available and low cost; the cross-linking reaction can be considered a green synthetic process since it takes place in water without the use of organic solvent or catalysts; and the resulting polymer is non-toxic and biodegradable.

Among the different water pollutants, the massive use of antibiotics for both human medicine and farming and the low efficiency of primary and secondary WWTP treatments to remove them is a concern. CIP is a prime example and, as a consequence of its zwitterionic nature, the sorption on **pSt** is pH-dependent and enhanced at alkaline pH, where CIP^−^ is the most abundant species and the interactions with **pSt** are strong and mainly independent of the ionic strength. At acidic pH, the predominant species is CIP^+^, and the interactions are weak and screened by ionic strength. These characteristics enable CIP desorption and **pSt** reuse following an environmentally friendly treatment with 20 mM AcONa pH 4.6. Beyond economic savings, the reuse of **pSt** is important from the perspective of waste reduction since it allows the removal of CIP before disposal.

The facts that **pSt**: (i) is obtained from affordable starting materials; (ii) is synthesized and regenerated by organic solvent-free procedures; (iii) shows high affinity for CIP in water, with an estimated Kd value of 1469 L/kg and a removal rate higher than 92% in the range 60–200 µg/L; (iv) traps CIP even at high concentration of NaCl, such as those in seawater or brine, and (v) its performance is improved at mild alkaline pH, validates our hypothesis and make **pSt** a promising material for field validation.

## Figures and Tables

**Table 1:** Isotherms that allow the non-linear fitting of the sorption of CIP and OFL on **pSt**.

Isotherm	CIP-pSt	OFL-pSt
Freundlich	K_F_ = 1.533 × 10^4^	K_F_ = 7.977 × 10^2^
	r.s.d. (%) = 41	r.s.d. (%) = 14
	N = 1.5303	N = 1.193
	r.s.d. (%) = 5.3	r.s.d. (%) = 2.8
	MWSE = 6.76 × 10^−3^	MWSE = 2.45 × 10^−3^
Temkin	K_1_ = 9.425 × 10^0^	K_1_ = 5.692 × 10^−1^
	r.s.d. (%) = 7.3	r.s.d. (%) = 9.8
	K_2_ = 5.664 × 10^1^	K_2_ = 3.139 × 10^1^
	r.s.d. (%) = 6.5	r.s.d. (%) = 8.2
	MWSE = 2.12 × 10^−2^	MWSE = 4.56 × 10^−2^

## Data Availability

Data will be made available on request.

## Supplementary Materials

The following supporting information can be downloaded at: https://www.mdpi.com/article/10.3390/polym15153188/s1, Figure S1: General setting for the fixed bed studies with **pSt**; Figure S2: DRX of the starting material β-CD, St and Dx; Figure S3: Characterization of the polymers **pSt**, **pDx** and **pCD-Dx** as sorbent for CIP and OFL; Figure S4: SEM micrograph of **pCD-Dx** at 10.00 K x; Figure S5: Derivative TGA of **pSt**; Figure S6: IR-TGA of **pSt**; Figure S7: Estimation of the sorption coefficient of CIP (a) and OFL (b) on **pSt**; Figure S8: Negative effect of the salinity on the sorption of CIP on **pSt** and improvement of the performance by buffering to pH 8; Figure S9: Fixed bed studies of the sorption of CIP on **pSt**; Table S1: Equations of the isotherm model assayed for the fitting of the experimental sorption data as defined by ISOT_calc; Table S2: Elemental analysis of cross-linked polymers; Table S3: Isotherm models that fail the non-linear fitting of the sorption of CIP and OFL on **pSt**. References [31,43] are cited in the supplementary materials.
