# Supplementary material for: Removal of the Water Pollutant Ciprofloxacin Using Biodegradable Sorbent Polymers Obtained from Polysaccharides
Source: Polymers (Basel). 2023 Jul 27;15(15):3188. doi: 10.3390/polym15153188 (PMC10421385; doi:10.3390/polym15153188)
Supplement: Supplementary file 1 [file polymers-15-03188-s001.zip › polymers-2494305-supplementary.pdf]

# Supplementary Materials

## Removal of the Water Pollutant Ciprofloxacin Using Biodegradable Sorbent Polymers Obtained from Polysaccharides

**Sarah Alvarado [1,†], Alicia Megia-Fernandez [1,2,3], Mariano Ortega-Muñoz [1,2,3], Fernando Hernandez-Mateo [1,2,3], F. Javier Lopez-Jaramillo [1,2,3,*] and Francisco Santoyo-Gonzalez [1,2,3,*]**

[1] Department Organic Chemistry, Faculty of Sciences, University of Granada, 18073 Granada, Spain
[2] Unit of Excellence in Chemistry Applied to Biomedicine and the Environment, University of Granada, 18073 Granada, Spain
[3] Biotechnology Institute, University of Granada, 18071 Granada, Spain
* Correspondence: fjljara@ugr.es (F.J.L.-J.); fsantoyo@ugr.es (F.S.-G.)
† Current address: Institute for Advanced Chemistry of Catalonia, CSIC, 08034 Barcelona, Spain.

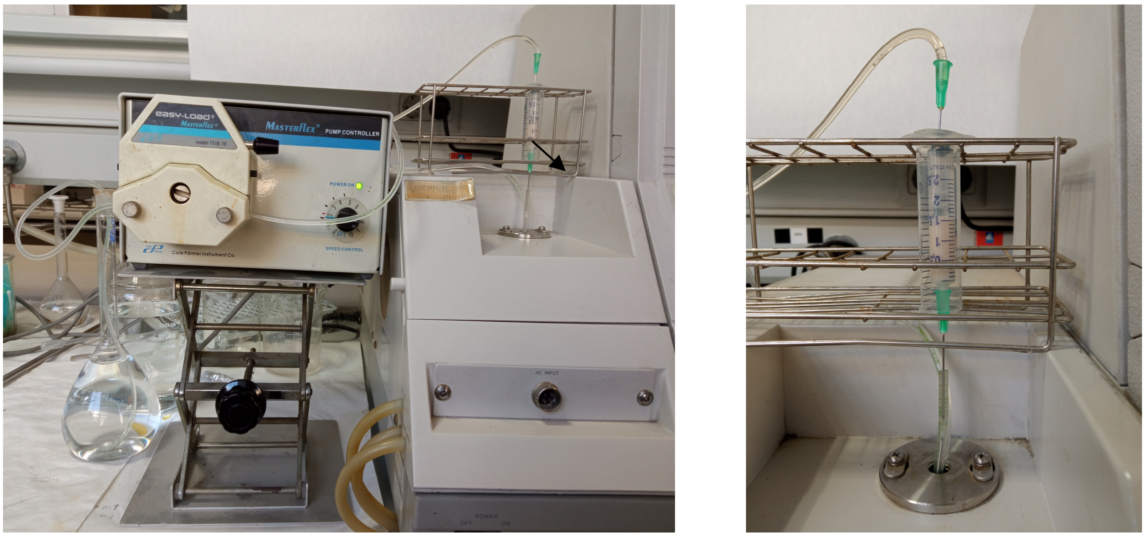


**Figure. S1**. (a) General setting for the fixed bed studies with **pSt**. (b) Detail of the **pSt** packing.

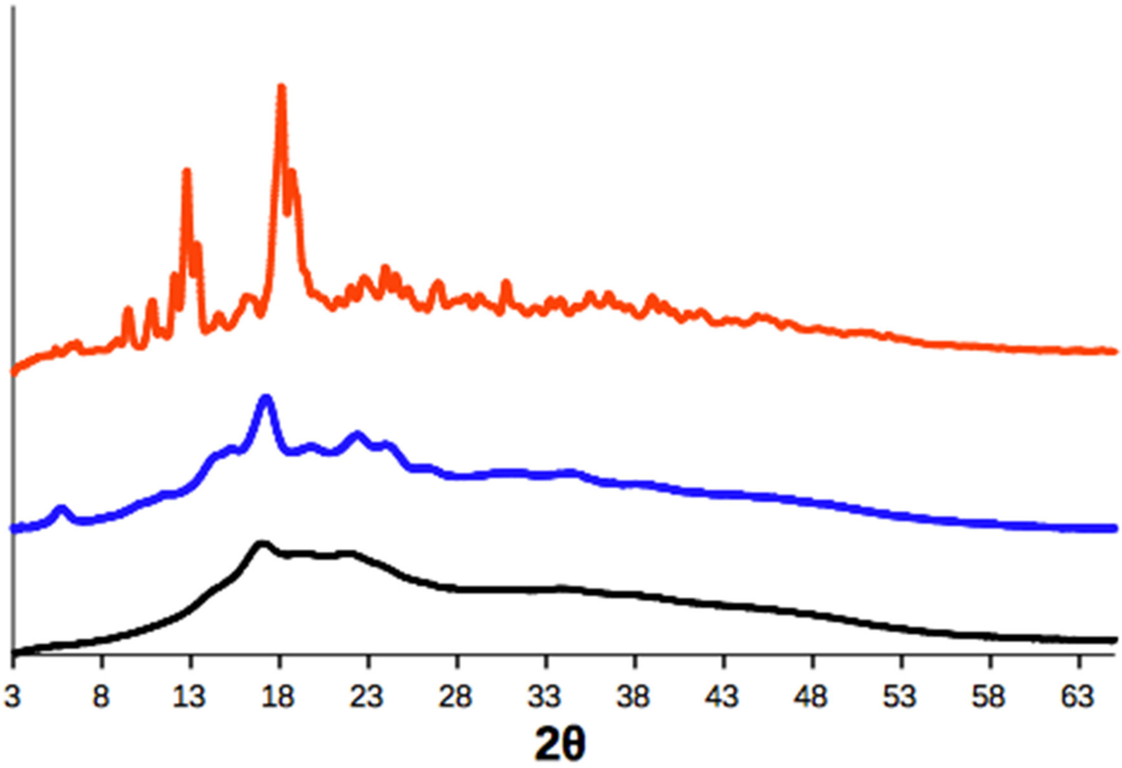


**Figure. S2.** DRX of the starting material β-CD (red) St (blue) and Dx (black) showing the d-spacing of some peaks.

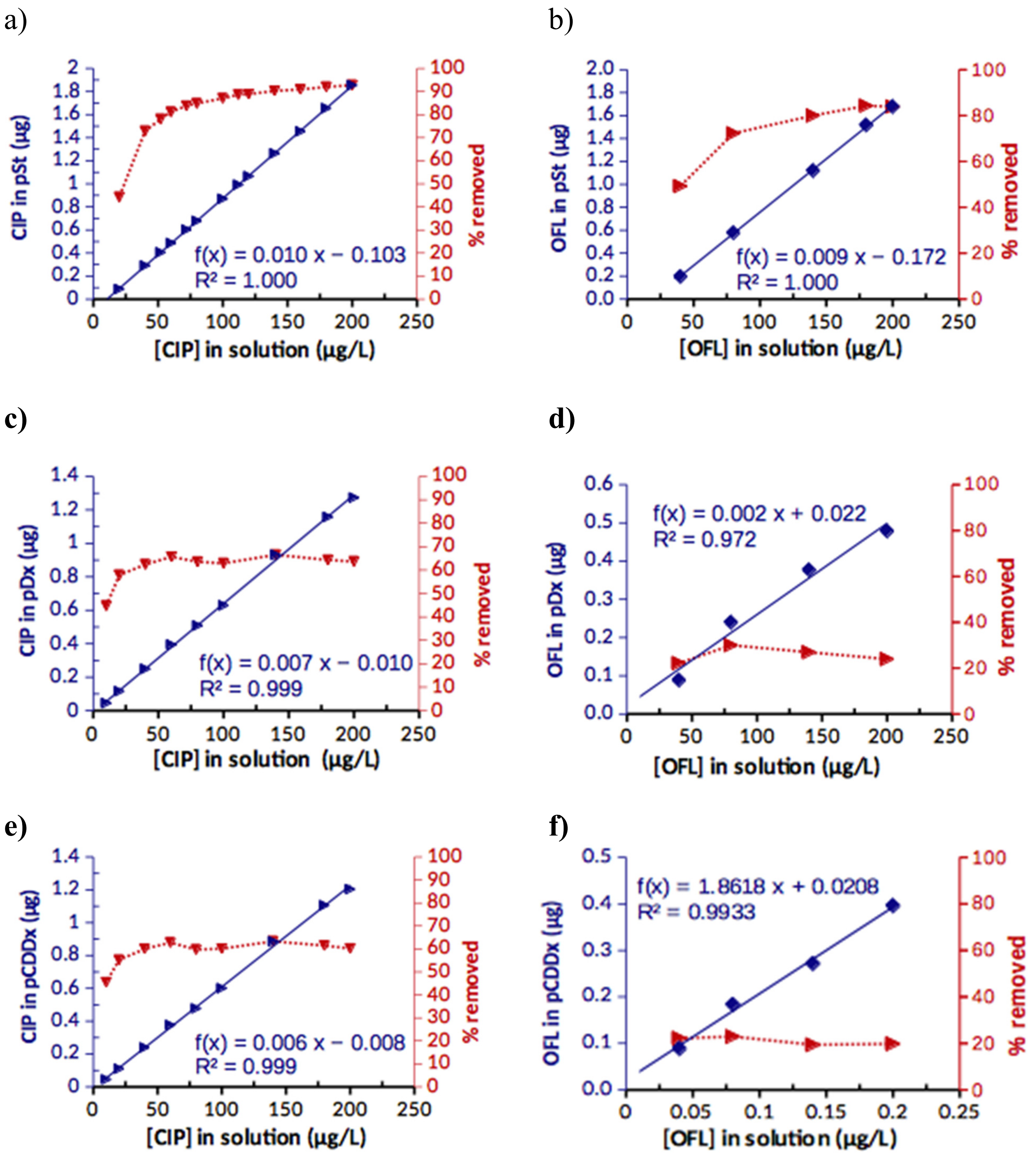


**Figure. S3.** Characterization of the polymers **pSt** (a & b), **pDx** (c & d) and **pCD-Dx** (e & f) as sorbent for CIP (a, c, e) and OFL (b, d, f). In blue, amount retained by the polymer and in red the percentage removed from the solution as a function of the initial concentration of quinolone.

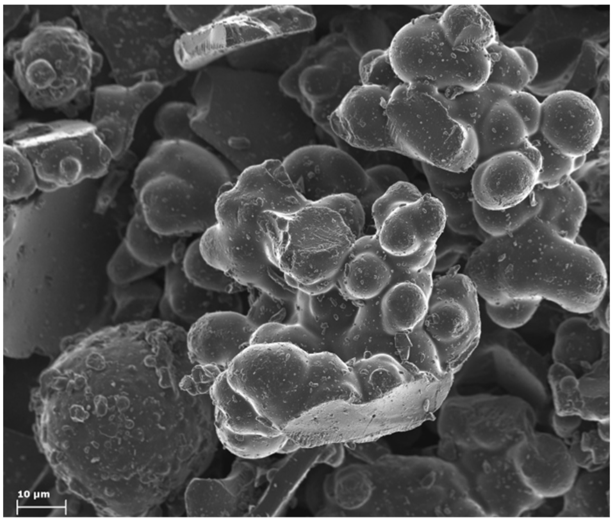


**Figure. S4.** SEM micrograph of **pCD-Dx** at 10.00 K x.

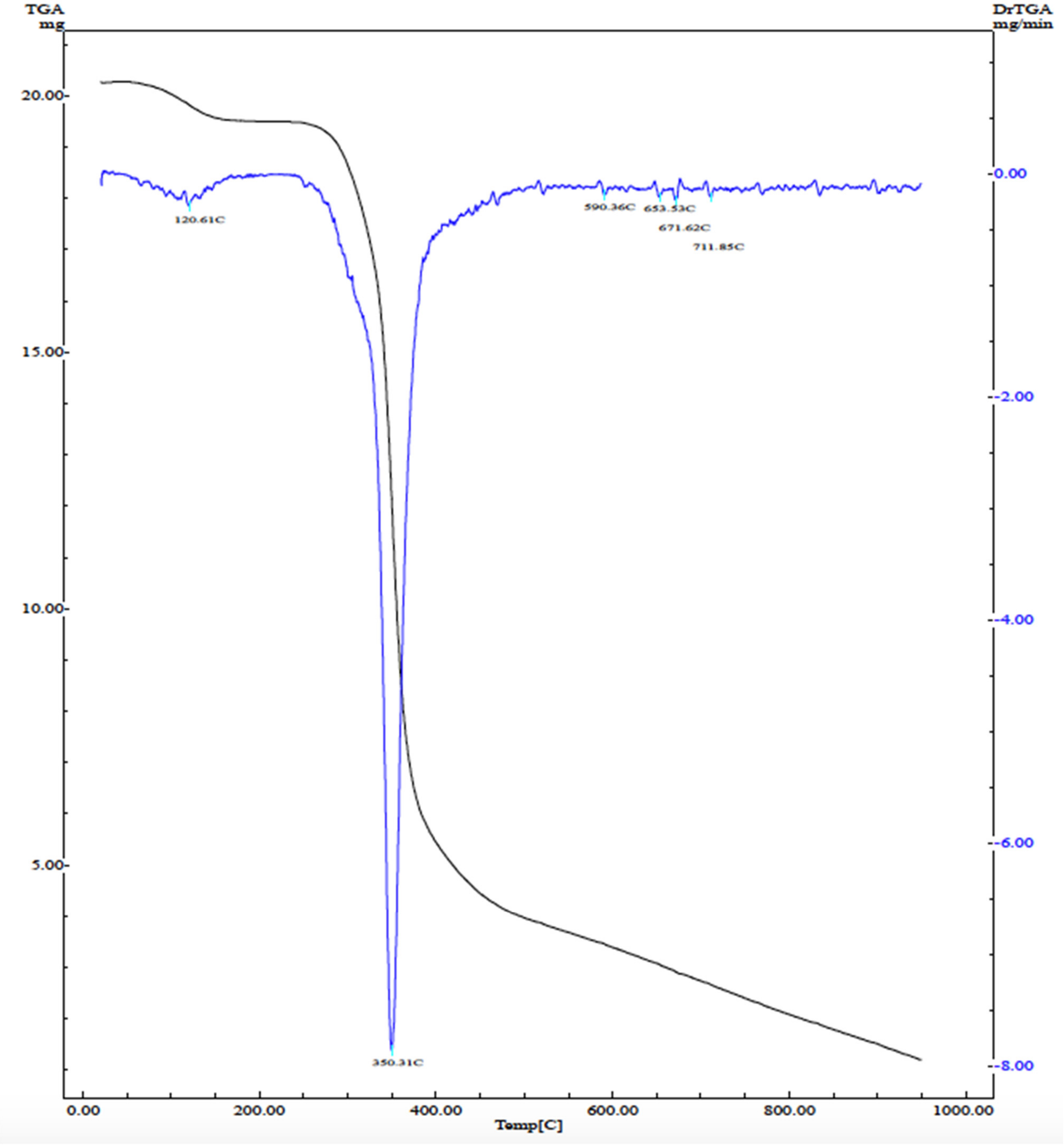


**Figure. S5** Derivative TGA of **pSt**. In black is shown the evolution of the mass of the sample as a function of the temperature and in blue the first derivative

**(a)**

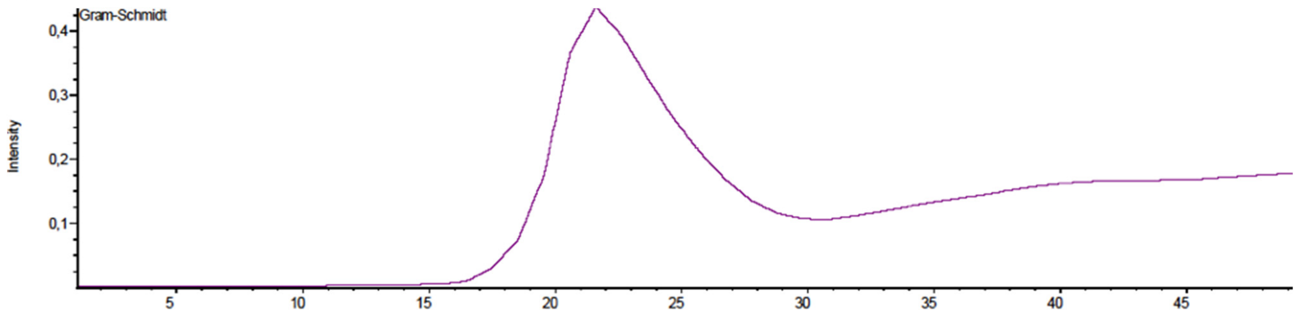


**(b)**

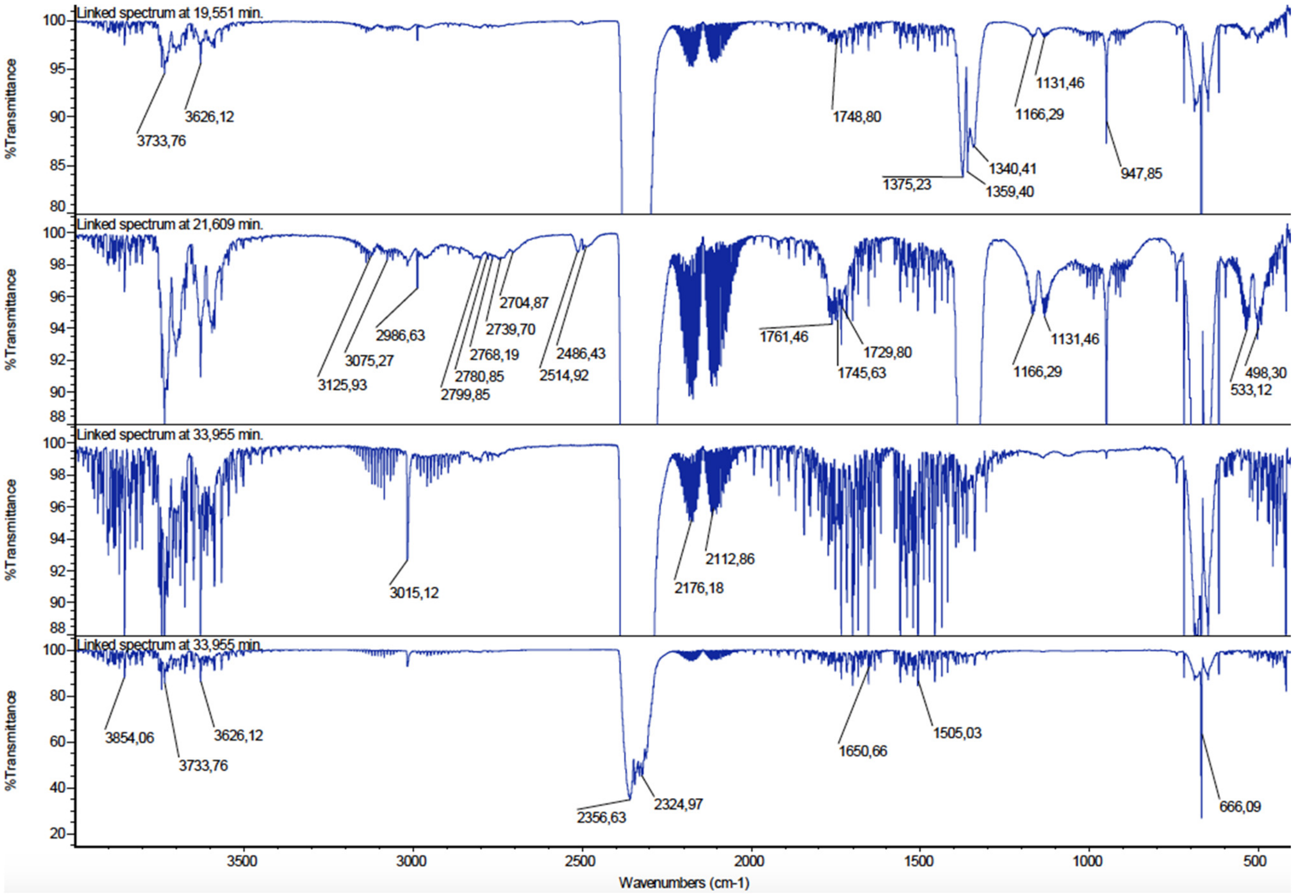


**Figure. S6.** IR-TGA of **pSt**. (a) Gram-Schmidt. (b) Most representative signals at three single time points 19.6 min, 21.6 min and 34.0 min. The delay between the TG and the FTIR detector is from 4 to 5 min.

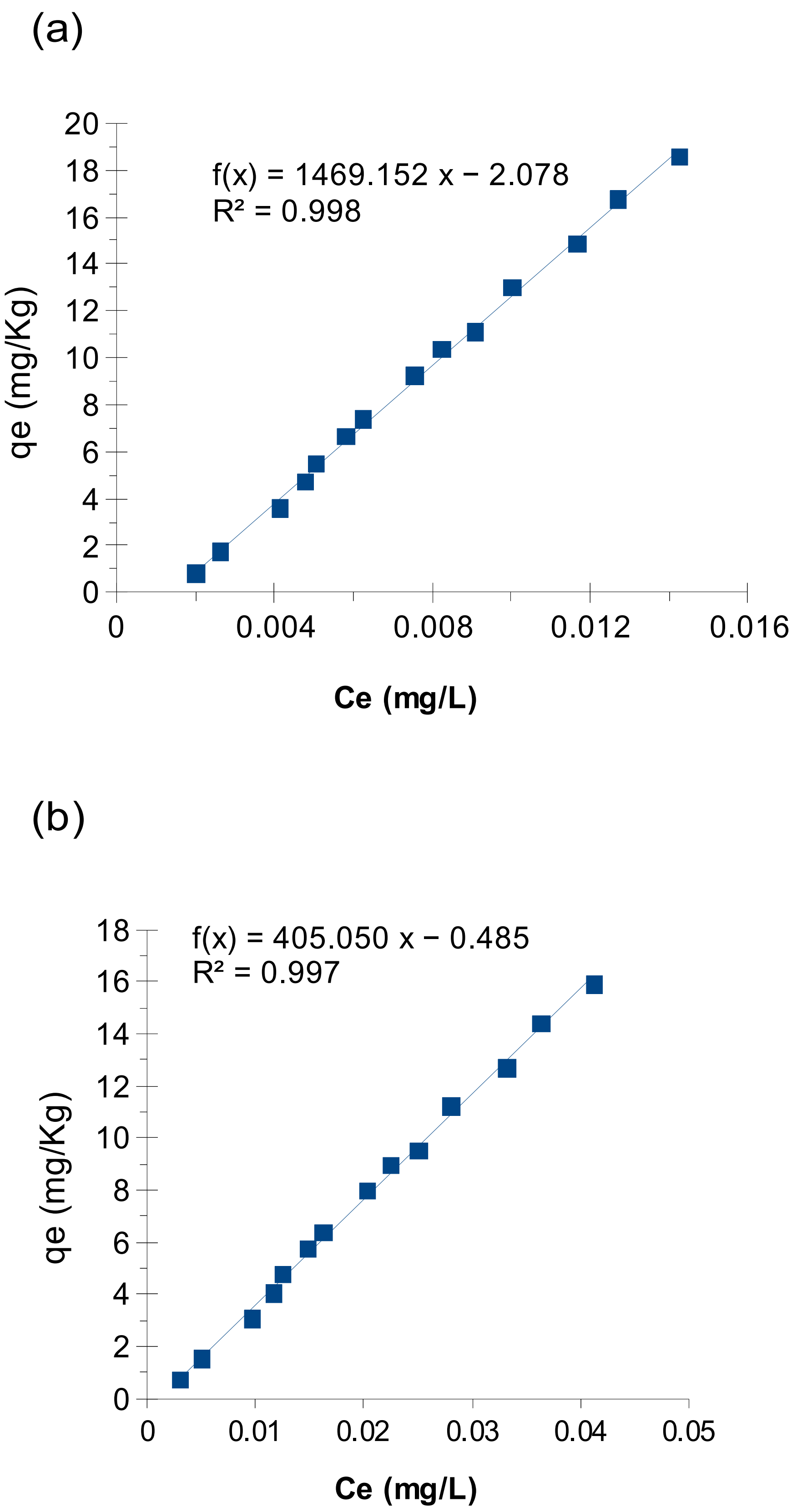


**Figure. S7.** Estimation of the sorption coefficient of CIP (a) and OFL (b) on **pSt**.

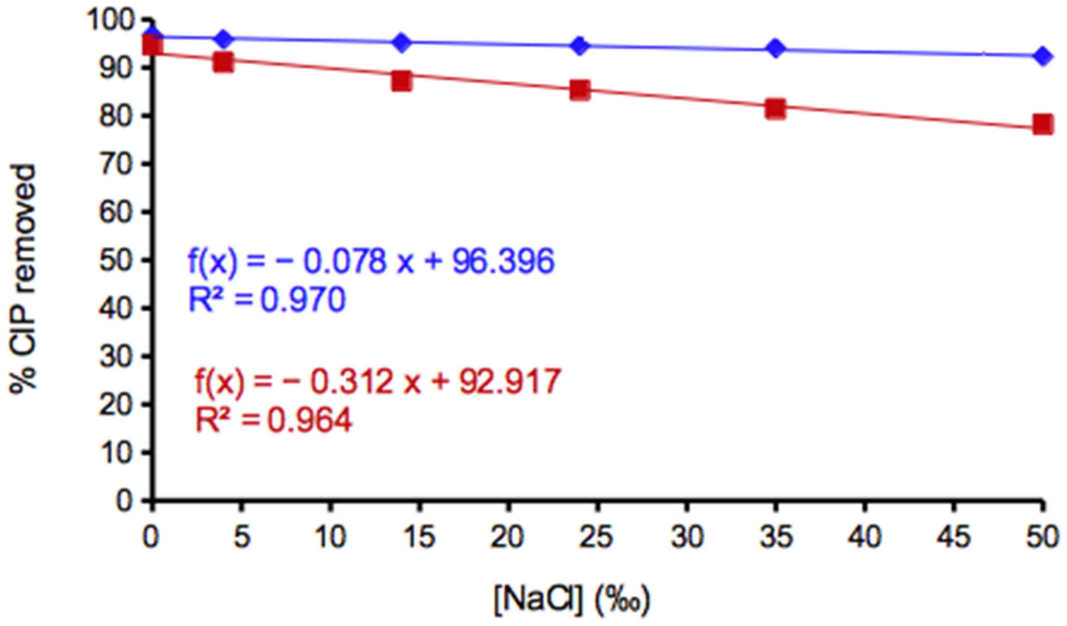


**Figure. S8.** Negative effect of the salinity on the sorption of CIP on **pSt** (red) and improvement of the performance by buffering to pH 8 (blue)

(a)

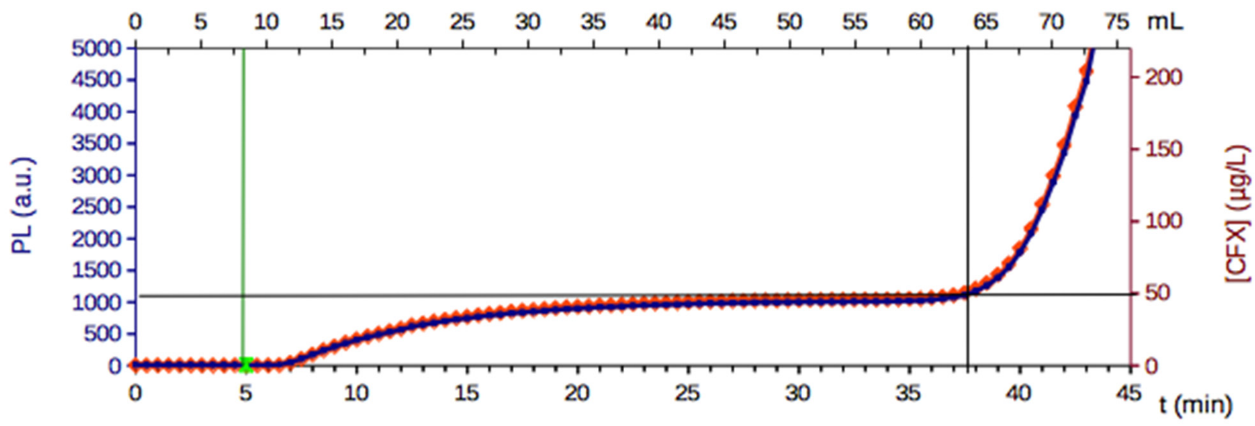


(b)

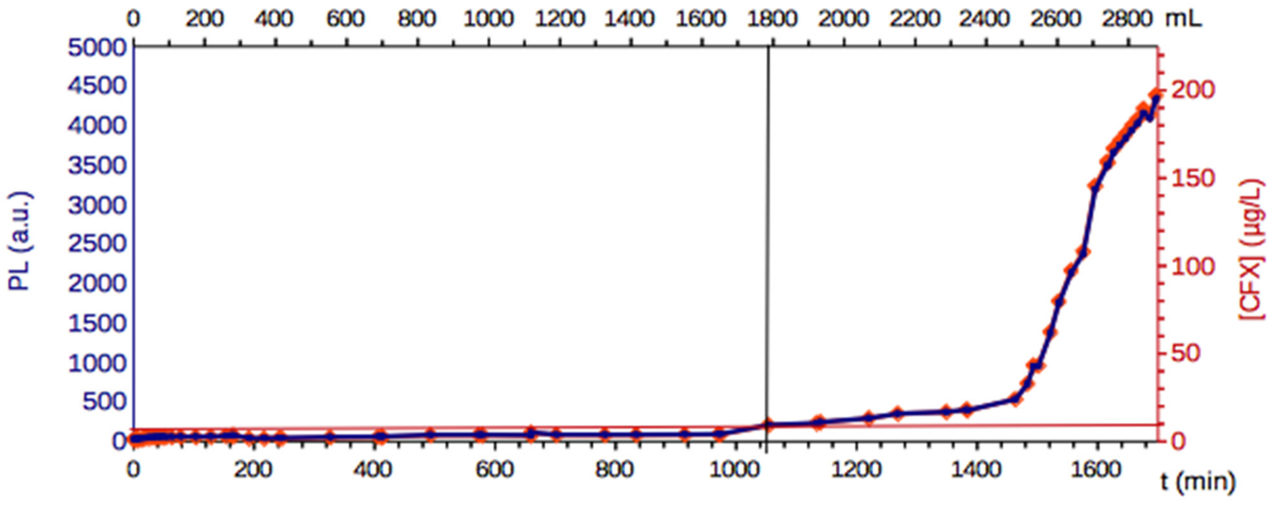


**Figure. S9.** Fixed bed studies of the sorption of CIP on **pSt**. Photoluminescence (arbitrary units, in blue) and concentration of CIP (µg/L, in red) of a solution of 10 mg/L (a) and 200 µg/L (b) flown through the regenerated **pSt** (0.5 g) packed into a 2.5 mL syringe. The green line in (a) marks the inition of pumping the solution of CIP.

**Table S1**. Equations of the isotherm model assayed for the fitting of the experimental sorption data as defined by ISOT_calc [31,43].

| **Isotherm (# parameters)** | **Equation ($q_e$ = ...)** | **Parameter definition** [43] |
|---|---|---|
| Langmuir (2) | $S_T \frac{K_L \cdot C_e}{1 + K_L \cdot C_e}$ | $S_T$ is maximum adsorption capacity<br>$K_L$ is affinity constant |
| Freundlich (2) | $K_F \cdot C_e^N$ | $K_F$ is adsorption potential<br>*N* is strength constant associated to heterogeneity (for homogenous systems N=1) |
| Temkin (2) | $k_1 \cdot \ln(C_e) + k_2$ | $k_1$ and $k_2$ are constants of Temkin isotherm and<br>$k_1$ is related to sorption heat |
| Vieth-Sladek (3) | $K_D \cdot C_e + \frac{S_T \cdot b \cdot C_e}{1 + b \cdot C_e}$ | $K_D$ and *b* are Vieth-Sladek constants<br>$S_T$ is the maximum adsorption capacity |
| Redlich-Peterson (3) | $S_T \frac{k \cdot C_e}{1 + (k \cdot C_e)^N}$ | $S_T$ is maximum adsorption capacity<br>*k* is related to constants of Redlich-Peterson model<br>*N* ranges from 0 to 1 |
| 2-sites Langmuir (4) | $\frac{S_{T1} \cdot k_1 \cdot C_e}{1 + k_1 \cdot C_e} + \frac{S_{T2} \cdot k_2 \cdot C_e}{1 + k_2 \cdot C_e}$ | $S_{T1}$ and $S_{T2}$are the maximum adsorption capacity for sites 1 and 2<br>$K_1$ and $K_2$ are affinity constant for sites 1 and 2 |

**Table S2.** Elemental analysis of cross-linked polymers. Results are expressed as means ± SD(n = 3)

| | **pSt** | **pDx** | **pCD-Dx** |
|---|---|---|---|
| **% C** | 41.04 ± 1.387 | 39.77 ± 0.381 | 40.13 ± 0.255 |
| **% H** | 7.45 ± 0.236 | 7.49 ± 0.307 | 7.81 ± 0.116 |
| **% S** | 7.16 ± 0.453 | 7.40 ± 0.080 | 7.51 ± 0.163 |

**Table S3.** Isotherm models that fail the non-linear fitting of the sorption of CIP and OFL on **pSt**.

| | Langmuir | Vieth-Sladek | Redlich-Petrson | 2 sites Langmuir |
|---|---|---|---|---|
| **CIP-pSt** | $S_T$=1.033x$10^3$<br>*r.s.d. (%)=1.6x$10^3$*<br>$K_L$=1.119x$10^{-0}$<br>*r.s.d. (%)=1.6x$10^3$*<br>*MWSE=4.82x$10^{-2}$* | $K_D$=1.144x$10^3$<br>*r.s.d. (%)=7.7x$10^0$*<br>$S_T$=1.000x$10^{-1}$<br>*r.s.d. (%)=2.6x$10^6$*<br>b=1.000x$10^0$<br>*r.s.d. (%)=2.6x$10^6$*<br>*MWSE=4.71x$10^{-2}$* | $S_T$=2.619x$10^3$<br>*r .s.d. (%)=1.8x$10^6$*<br>K=8.733x$10^{-1}$<br>*r.s.d. (%)=1.8x$10^6$*<br>N=1.000x$10^0$<br>*r.s.d. (%)=2.6x$10^6$*<br>*MWSE=4.71x$10^{-2}$* | $S_{T1}$=1.038x$10^3$<br>*r.s.d. (%)=1.8x$10^3$*<br>$K_{L1}$=1.114x$10^0$<br>*r.s.d. (%)=1.8X$10^3$*<br>$S_{T2}$=1.020x$10^3$<br>*r.s.d. (%)=2.7x$10^6$*<br>$K_{L2}$=1.114x$10^{-3}$<br>*r.s.d. (%)=2.7x$10^6$*<br>*MWSE=4.82x$10^{-2}$* |
| **OFL-pSt** | $S_T$=5.468x$10^5$<br>*r.s.d. (%)=1.2x$10^5$*<br>$K_L$=6.381x$10^{-4}$<br>*r.s.d. (%)=1.2x$10^5$*<br>*MWSE=1.29x$10^{-2}$* | $K_D$=3.666x$10^2$<br>*r.s.d. (%)=4.3x$10^0$*<br>$S_T$=1.000x$10^0$<br>*r.s.d. (%)=1.3x$10^6$*<br>b=1.000x$10^0$<br>*r.s.d. (%)=1.3x$10^6$*<br>*MWSE=1.14x$10^{-2}$* | $S_T$=1.023x$10^3$<br>*r.s.d. (%)=8.9x$10^5$*<br>K=7.164x$10^{-1}$<br>*r.s.d. (%)=8.9x$10^5$*<br>N=1.000x$10^{-0}$<br>*r.s.d. (%)=1.3x$10^6$*<br>*MWSE=1.14x$10^{-2}$* | $S_{T1}$=5.605x$10^5$<br>*r.s.d. (%)=5.1x$10^5$*<br>$K_{L1}$=6.540x$10^{-4}$<br>*r.s.d. (%)=5.1X$10^5$*<br>$S_{T2}$=5.468x$10^3$<br>*r.s.d. (%)=1.3x$10^6$*<br>$K_{L2}$=6.381x$10^{-4}$<br>*r.s.d. (%)=1.3x$10^6$*<br>*MWSE=1.14x$10^{-2}$* |

## References

31. Beltrán, J.L.; Pignatello, J.J.; Teixidó, M. ISOT_Calc: A Versatile Tool for Parameter Estimation in Sorption Isotherms. *Comput. Geosci.* **2016**, *94*, 11–17. https://doi.org/10.1016/j.cageo.2016.04.008.

43. Majd, M.M.; Kordzadeh-Kermani, V.; Ghalandari, V.; Askari, A.; Sillanpää, M. Adsorption Isotherm Models: A Comprehensive and Systematic Review (2010−2020). *Sci. Total Environ.* **2022**, *812*, 151334. https://doi.org/10.1016/j.scitotenv.2021.151334.
